# Epidemiology of bee stings in Campina Grande, Paraíba state, Northeastern Brazil

**DOI:** 10.1186/1678-9199-20-13

**Published:** 2014-04-02

**Authors:** Ana Thaise Sousa Linard, Rafaella Moreno Barros, Jorge Alves Sousa, Renner Souza Leite

**Affiliations:** 1 Health Academic Unit, Education and Health Center, Federal University of Campina Grande, Cuité campus, Cuité, Paraíba State, Brazil; 2 Education Academic Unit, Education and Health Center, Federal University of Campina Grande, Cuité campus, Cuité, Paraíba State, Brazil

**Keywords:** Africanized honeybee, Paraíba, Human envenomation, Venomous animals, Public health

## Abstract

**Background:**

The present study aims to investigate the clinical-epidemiological characteristics of bee sting cases recorded between 2007 and 2012 in the city of Campina Grande, Paraíba state, Brazil. Data were collected from the database of the Injury Notification Information System of the Brazilian Ministry of Health.

**Results:**

A total of 459 bee sting cases were retrospectively analyzed. The average annual incidence was 19 cases per 100,000 inhabitants. Cases were distributed in all months of the year, with higher prevalence in September and February. Most victims were men aged between 20 and 29 years. The highest incidence of cases was recorded in urban areas. Victims were stung mainly on the head and torso and received medical assistance predominantly 1 to 3 hours after being stung. The most frequent clinical manifestations were pain, edema and itching. Most cases were classified as mild, and three deaths were reported.

**Conclusions:**

The high incidence of envenomations provoked by bees in Campina Grande suggests that it may be an important risk area for accidents. Since several medical records lacked information, clinical-epidemiological profile of bee sting cases in the studied region could not be accurately determined.

The current study provides relevant data for the development of strategies to promote control and prevention of bee stings in this area. Further training for health professionals seems to be necessary to improve their skills in recording clinical-epidemiological information as well as in treating bee sting victims.

## Background

Venomous animals comprise a significant health problem in many parts of the world, particularly in Africa, Southeast Asia and Tropical America. In these regions, epidemiological studies on venomous animals are generally restricted to envenomation by snakes and scorpions. Therefore, little information regarding other venomous animals is available [1]. In Latin America, bee stings are considered a public health problem due to the high incidence and clinical severity of such cases [2,3]. The incidence of bee stings, regardless of the species involved, varies from country to country and between regions in a country, depending on diverse factors including climate, ecological parameters, biodiversity, distribution of species, human population density, economic activities, types of dwellings, among others.

Bee envenomation may induce two distinct clinical manifestations, depending on the victim’s sensitivity to the venom and the number of stings [4,5]. These manifestations may be toxic reactions, attributed to the pharmacological action of the venom, or allergic reactions, in which the mechanisms of hypersensitivity are involved [3]. A person may be affected by one or a few stings. In these cases, symptoms vary from mild local inflammatory reactions to severe allergic reactions, which may lead to anaphylactic shock. When the victim is attacked by swarms, thus suffering multiple stings, severe systemic toxic manifestations occur as a result of the greater amount of venom inoculated [3,4].

African honeybees (*Apis mellifera scutellata*), characterized by their aggressive nature and high honey production, were introduced in Brazil in 1956. In the following year, there was an accidental release of some queens of this species, resulting in the hybridization or africanization of European bees (*Apis mellifera mellifera* and *Apis mellifera ligustica*) and uncontrolled breeding of the Africanized species in the Brazilian environment. The frequency of serious and/or fatal injuries caused by such bees has increased since then [5,6]. According to the Brazilian Health Ministry, an expressive increase in the number of bee sting cases have been observed in the last ten years [7]. The absence of antivenom makes this problem even more complex, thus reinforcing the importance of having an effective treatment.

In 2011, approximately 9,447 cases were registered in Brazil, with an incidence of 4.9/100,000 inhabitants and 26 deaths. The South region has the highest incidence (7.9/100,000 inhabitants), followed by the Northeast (5.3/100,000 inhabitants), Southeast (4.5/100,000 inhabitants), Central-West (3.0/100,000 inhabitants) and North (2.3/100,000 inhabitants) [7]. Bee sting cases appear to be more frequent in urban areas and affect more frequently male individuals aged between 20 and 49 years, who are predominantly stung on the head and torso [3].

Information on the incidence of bee sting cases, lethality rate, clinical manifestations and progression of the cases are essential for a more clear evaluation of the problem, as well as for improving medical care for victims and developing public policies aimed at reducing the incidence of envenomation. Despite the medical importance regarding bee stings and the increasing number of cases registered in the Northeast of Brazil, the epidemiology of bee stings in this region is little known. In order to fill this gap, we investigated the clinical-epidemiological profile of bee sting cases recorded from 2007 to 2012 in the city of Campina Grande, Paraíba state, Northeastern Brazil.

## Methods

### Study area

**Figure 1:**
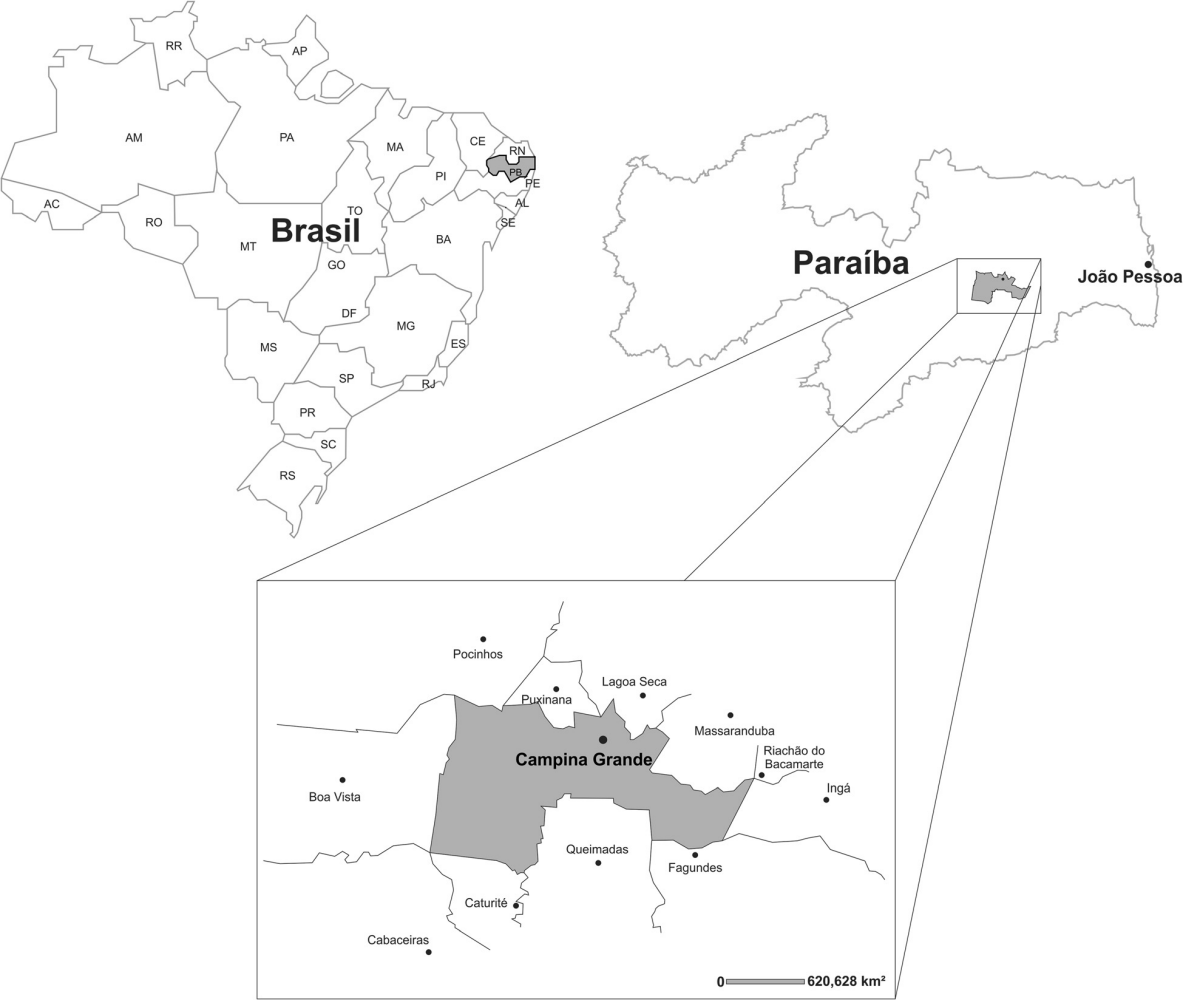
Studied area of bee sting cases in Paraíba state, Brazil.

This study took place in the city of Campina Grande, located in the countryside of the state of Paraíba, Northeastern Brazil, at the approximate geographical coordinates 07° 13′ 50′ S and 35° 52′ 54″ W (Figure 1). This region is at an altitude of 555 meters above sea level, presenting tropical altitude climate, with an average annual temperature of 25°C [8]. Its population is estimated to be 385,276 inhabitants, covering a geographical area of 599.6 km^2^, with demographic density of 597.9 inhabitants/km^2^[9].

### Data acquisition

Medical records of bee sting cases that occurred in the city of Campina Grande from 2007 to 2012 were retrospectively analyzed. Data were collected from the Third Health Sector of Campina Grande, using the database of the Ministry of Health Injury Notification Information System (SINAN). Epidemiological variables were analyzed according to:

• gender and age of the victim at the time of the accident;

• sting site on the victim’s body;

• month and year of the occurrence;

• zone (urban or rural) of the occurrence;

• time elapsed between the accident and medical assistance.

Variables investigated during the clinical evaluation were local and systemic manifestations, as well as severity and progression of the cases. Demographic and population data provided by the Brazilian Institute of Geography and Statistics (IBGE) were used to calculate incidence rates. Statistical analyses were performed by means of simple frequency tests, using the SPSS® (Statistical Package for Social Sciences) software, 13.0 version for Windows. This research was approved by the Ethics Research Committee of Alcides Carneiro University Hospital of the Federal University of Campina Grande (protocol number 146.431), following the guidelines established by the Declaration of Helsinki.

## Results

A total of 459 bee sting cases were registered from January 2007 to December 2012 in the city of Campina Grande, with an average incidence rate of 28 cases per 100,000 inhabitants in 2007, 27 cases per 100,000 inhabitants in 2008, 24 cases per 100,000 inhabitants in 2009, 10 cases per 100,000 inhabitants in 2010, 13 cases per 100,000 inhabitants in 2011, and 14 cases per 100,000 inhabitants in 2012.

Table 1 shows that bee sting cases were registered in all months of the year, with higher incidence in the third trimester (n = 146; 31.90%). Table 2 shows the individual characterization and circumstances of the occurrences. Victims were predominantly male (n = 311; 68.2%) and aged between 20 and 29 years (n = 120; 26.14%). Most cases occurred in urban areas (n = 356; 77.55%), with victims who were not at work (n = 394; 85.83%). The most affected area of the victim’s body was the head (n = 152; 33.11%), and victims received medical assistance up to one hour after being stung (n = 101; 22%). Table 3 shows the severity and progression of the cases. The majority of cases was classified as mild (n = 373; 81.26%), some cases as moderate (n = 61; 13.28%), and a few cases as severe (n = 12; 2.61%). Most cases progressed to cure (n = 423; 92.15%), and three deaths were registered. Table 4 shows local and systemic clinical manifestations resulting from envenomation. The most frequent local manifestations were pain (n = 410; 89.32%), edema (n = 295; 64.27%) and itching (n = 14; 3.05%), whereas the most frequent systemic clinical symptoms were vagal (n = 20; 4.35%), neurological (n = 6; 1.3%) and myolytic manifestations (n = 2; 0.43%).

**Table 1:** Monthly distribution of bee sting cases in Campina Grande, Paraíba state, between 2007 and 2012 (n = 459)

**Month**	**Year**						**TOTAL**	
	**2007**	**2008**	**2009**	**2010**	**2011**	**2012**	**n**	**%**
January	9	7	5	12	1	5	39	8.5%
February	4	6	14	6	15	7	52	11.4%
March	10	9	7	4	0	5	35	7.6%
April	8	4	18	1	0	11	42	9.2%
May	5	5	0	6	0	5	21	4.6%
June	4	10	10	0	2	0	26	5.7%
July	15	11	4	6	2	4	42	9.2%
August	19	12	5	1	6	5	49	10.7%
September	15	10	14	0	9	7	55	12%
October	5	9	7	0	10	1	33	7.2%
November	6	17	5	0	4	0	32	7%
December	7	4	7	6	2	6	33	7.2%
**TOTAL**	107	104	96	42	51	56	459	100%

**Table 2:** Descriptive analyses of bee sting cases in Campina Grande, Paraíba state, between 2007 and 2012, according to epidemiological variables (n = 459)

**Variable**	**Year**						**TOTAL**	
	**2007**	**2008**	**2009**	**2010**	**2011**	**2012**	**n**	**%**
**Age group (years)**								
1∣—∣9	14	17	13	5	8	4	61	13.3%
10∣—∣19	16	17	13	6	14	11	77	16.8%
20 ∣—∣ 29	28	31	30	14	8	9	120	26.1%
30 ∣—∣39	18	14	18	7	7	13	77	16.8%
40 ∣—∣ 49	13	15	10	8	5	3	54	11.8%
50 ∣—∣ 59	10	3	6	2	6	6	33	7.2%
60 ∣—∣ 69	5	2	4	1	1	4	17	3.7%
≥70	3	5	2	2	2	6	20	4.3%
Unknown	0	0	0	0	0	0	0	0%
**Gender**								
Male	71	72	72	32	26	38	311	68.2%
Female	36	32	24	13	25	18	148	32.4%
**Place of occurrence**								
Urban	82	77	81	35	42	39	356	77.6%
Rural	23	26	11	7	7	16	90	19.6%
Peri-urban	0	0	0	2	0	0	2	0.4%
Unknown	2	1	4	1	2	1	11	2.4%
**Work-related accident**								
Yes	9	12	5	2	5	9	42	9.2%
No	85	90	86	43	45	45	394	85.8%
Unknown	13	2	5	0	1	2	23	5%
**Part of the body stung**								
Head	32	37	35	13	15	20	152	34.1%
Torso	11	20	14	7	7	8	67	15%
Hand	13	6	9	2	6	4	40	9%
Foot	10	5	8	2	1	13	39	8.7%
Arm	6	6	6	4	3	6	31	7%
Forearm	3	1	0	0	1	3	8	1.8%
Leg	0	2	3	1	1	1	8	1.8%
Unknown	29	24	18	13	16	1	101	22.6%
**Time elapsed between sting and assistance (hours)**								
0 ∣—∣1	28	26	18	8	8	13	101	22%
1 ∣—∣ 3	17	21	13	8	9	11	79	17.2%
3 ∣—∣ 6	9	5	9	2	1	3	29	6.3%
6 ∣—∣ 12	4	1	4	0	3	0	12	2.6%
≥12	13	20	9	8	8	5	63	13.8%
Unknown	36	31	43	19	22	24	175	38.1%
**TOTAL**	107	104	96	45	51	56	459	100%

**Table 3:** Severity and progression of bee sting cases in Campina Grande, Paraíba state, between 2007 and 2012 (n = 459)

	**Severity**				**Progression**		
	**Mild**	**Moderate**	**Severe**	**Unknown**	**Cured**	**Unknown**	**Death**
**TOTAL**	373 (81.3%)	61 (13.3%)	12 (2.6%)	13 (2.8%)	423 (92.1%)	33 (7.2%)	3 (0.7%)

**Table 4:** Clinical symptoms of bee sting victims in Campina Grande, Paraíba state, between 2007 and 2012 (n = 459)

**Clinical symptoms**					
**Local symptoms**	**n**	**%**	**Systemic symptoms**	**n**	**%**
Pain	410	55.2%	Vagal manifestations	20	66.7%
Edema	295	39.8%	Neurological manifestations	6	20%
Itching	14	1.9%	Myolytic manifestations	2	6.7%
Paresthesia	7	1%	Pain	1	3.3%
Ecchymosis	7	1%	Arterial hypotension	1	3.3%
Erythema	7	1%			
Headache	1	0.1%			
Necrosis	1	0.1%			

## Discussion

In South and Central American countries, epidemiological data on the incidence of bee sting cases are scarce and incomplete, probably due to underreporting of cases and a deficiency in recording clinical and epidemiological data [2,3,10]. Moreover, mortality records are incomplete and a few autopsies have been done on bee sting victims [10]. Because of the lack of medical statistics, it is difficult to establish the severity of bee sting cases and even more difficult to estimate the seriousness of this problem in the future.

According to the Brazilian Ministry of Health, bee stings are more frequent in the Southeast region, followed by the Northeast. In the Northeast, 11,753 bee sting cases were recorded between 2000 and 2011, with 43 reports of death [7]. Currently, among the northeastern states, Pernambuco has the highest prevalence, followed by the states of Bahia, Rio Grande do Norte and Ceará, respectively [7]. In Paraíba, 611 bee sting cases were recorded in the period from 2000 to 2012, with only two deaths [7].

The current study shows that 459 bee sting cases were recorded in Campina Grande between 2007 and 2012, representing an average of 76 cases per year, and an annual incidence of 19 cases per 100,000 inhabitants. These values are significant, especially when compared with the number of cases recorded in the whole state of Paraíba between 2006 and 2011. The high incidence of stings in Campina Grande suggests that it may be an important risk area for such injury. In Brazil, Africanized honeybees found in the northeast differ dramatically from the species found in the southeast, in their sensitivity to disturbance, their ability to communicate alarm within and among colonies, and their capacity to respond quickly by massive and persistent attack on intruders [3,11]. The availability of pollen, nectar and water, as well as the anthropogenic interference, increase the mobility of bees in the environment, thus bringing them closer to humans and favoring their attacks [12,13]. These factors may explain, at least in part, the high incidence of bee envenomation in the studied region.

Bee stings were recorded throughout the year, with a slight increase in frequency between July and September (n = 146; 31.9%), suggesting a seasonal distribution of cases. In Paraíba and other states of the Northeastern Brazil, this period coincides with the rainy season, when there is great offer of food – mainly determined by the flowering of herbaceous and bush plants [11,13]. The little variation in the monthly distribution of cases may be attributed to the climatic conditions of the region, which are characterized by defined seasons and an average annual temperature of 25°C [8].

Our findings suggest that preventive actions against bee stings should be carried out throughout the year and intensified during the months of higher incidence. The risk of bee stings could be reduced by mapping and removal of hives, as well as development of educational campaigns aimed at improving knowledge of the general population, beekeepers and specialized health workers about preventive measures against envenomation by bees. It would be also important to promote training for beekeepers on the management of hives.

Bee stings were more frequent in male individuals (n = 311; 68.2%), indicating differential risk between men and women in the region. This result corroborates an epidemiological observation on bee stings carried out in the state of Santa Catarina [14]. Regarding victims’ age, most of them were between 20 and 29 years old (n = 120; 26.14%), followed by those aged between 30 and 39 years (n = 77; 16.77%). These findings indicate that bee sting cases occurred mostly in the economically active population.

Most cases occurred in urban areas (n = 356; 77.55%), which may be explained by the frequent installation of hives in human dwellings [13]. The presence of colonies in dwellings might also reflect some degree of synanthropy, since bees have clearly adapted to the conditions imposed by the cities, that is, smaller green areas, numerous buildings and high noise pollution. The great number of plants used in the afforestation of cities, with periods of flowering fairly distributed during the year, may contribute to the migration of bees from rural to urban areas. In 180 cases (39.2%), victims received medical assistance within three hours after being stung, and in 152 cases (33.11%) the head was the main affected area. However, these numbers are probably underestimated, since several medical records lacked information on both the time elapsed between sting and medical assistance (n = 175; 38.12%) and the affected body part (n = 101; 22%).

The majority of cases was classified as mild (n = 373; 81.26%); most cases progressed to cure (n = 423; 92.15%) and no patients had sequelae. Taken together, these findings suggest that bee envenomation may be provoked by a single or a few stings. The high frequency of victim recovery, as well as the lack of sequelae, might suggest that the public health system in this region is efficient. However, it is sensible to consider the possibility that there are few allergic persons among this specific population and that the recorded cases coincided with the non-severe ones. The most frequent local manifestations were pain (n = 410; 89.32%), edema (n = 295; 64.27%) and itching (n = 14; 3.05%). Typically, bee sting symptoms comprise local pain and edema, without systemic reactions. These reactions occur in all envenomed individuals to some degree and are caused by vasoactive components of bee venom rather than by an allergic mechanism [4]. The most frequent systemic clinical manifestations were vagal (n = 20; 4.35%) and neurological (n = 6; 1.30%) symptoms. These symptoms were observed to be the same as those found in other studies [5,15].

Three deaths were recorded, but their cause was not informed. This finding suggests that data from the Brazilian Ministry of Health are underestimated, since it informed only two cases in a ten-year period, whereas we identified three deaths in only six years. The cases resulting in death occurred with three male individuals in the rural area: one of them was a child, one was an adult and one was an elderly. In these three cases, the time elapsed between sting and medical care was higher than three hours, suggesting that the delay in medical assistance may have contributed for the severity of the cases. Moreover, it shows a close association between severity and amount of venom inoculated, the body mass and the patient’s sensitivity to the venom. It was not possible to distinguish between deaths attributed to allergic reactions and those attributed to envenoming. The clinical records of the Brazilian Ministry of Health do not precise whether the envenoming is caused by a single sting or multiple stings. It would be important to incorporate the distinction between allergic reactions and envenomation in the clinical records in order to have a more appropriate evaluation. This action might improve the treatment of victims.

Since clinical-epidemiological data are fundamental to improve knowledge on bee stings at regional levels, the enhancement of data collection procedures seems to be urgent [3-5,14-16]. Accordingly, a further understanding of bee sting epidemiology in the studied region seems to require better training for health workers and precise protocols for recording victim’s information. Without basic infrastructure and training, it will be difficult to gather precise evidence about health problems related to bee stings in the northeastern Brazil.

## Conclusions

The high incidence of bee sting cases suggests that this region may be a risk area in the Northeastern Brazil. Since several medical records lacked information, clinical-epidemiological profile of bee stings could not be accurately determined. Despite that, the current study may provide relevant data for the development of strategies to promote control and prevention of bee stings in the studied region. Further training for health professionals seems to be necessary to improve their skills in recording clinical-epidemiological information, as well as in treating bee sting victims.

### Ethics committee approval

The present study was approved by the Ethics Research Committee of Alcides Carneiro University Hospital of the Federal University of Campina Grande (protocol number 146.431), following the guidelines established by the Declaration of Helsinki.

## Competing interests

The authors declare that there are no competing interests.

## Authors’ contributions

LATS and BRM worked in the survey of the literature, in the research of data. SJA worked on statistical analysis of data and LRS worked on the conception and design the study, analysis and interpretation data, wrote the first version of the article. All authors read and approved the final manuscript.
